# Asymptomatic *Plasmodium* Infections in Children in Low Malaria Transmission Setting, Southwestern Uganda^1^

**DOI:** 10.3201/eid2208.160619

**Published:** 2016-08

**Authors:** Michelle E. Roh, Caesar Oyet, Patrick Orikiriza, Martina Wade, Gertrude N. Kiwanuka, Juliet Mwanga-Amumpaire, Sunil Parikh, Yap Boum

**Affiliations:** University of California, San Francisco, California, USA (M.E. Roh);; Yale School of Public Health, New Haven, Connecticut, USA (M.E. Roh, M. Wade, S. Parikh);; Mbarara University of Science and Technology, Mbarara, Uganda (C. Oyet, G.N. Kiwanuka, Y. Boum II);; Médecins Sans Frontières Epicentre Mbarara Research Centre, Mbarara (P. Orikiriza, J. Mwanga-Amumpaire, Y. Boum II)

**Keywords:** malaria, protozoan infections, parasitic diseases, Plasmodium, microscopy, children, asymptomatic infections, mosquitoes, mosquito nets, microscopy, PCR, Uganda, Africa, parasite

## Abstract

A survey of asymptomatic children in Uganda showed *Plasmodium malariae* and *P. falciparum* parasites in 45% and 55% of microscopy-positive samples, respectively. Although 36% of microscopy-positive samples were negative by rapid diagnostic test, 75% showed *P. malariae* or *P. oval*e parasites by PCR, indicating that routine diagnostic testing misses many non–*P. falciparum* malarial infections.

Since 2000, substantial progress has been made in reducing malaria worldwide. In Uganda, malaria transmission is heterogeneous, yet 97% of all cases are attributed to *P. falciparum* (*1*). Accordingly, detection and treatment algorithms have targeted *P. falciparum* over less virulent species. Inadequate attention to non–*P. falciparum* infections has several implications for malaria transmission. For example, gametocytemia can occur earlier (e.g., *P. vivax* and perhaps *P. ovale*) and remain undetected for longer periods because of milder clinical symptoms (e.g., *P. malariae* and *P. ovale*) than for *P. falciparum* infections, enabling persistent transmission of non–*P. falciparum* infections (*2*).

In the southwestern region of Uganda, *Plasmodium* transmission is low and unstable. In 2004 and 2010, we conducted surveys that showed progress in decreasing *P. falciparum* infections in this region, although comparatively little is known about the prevalence of other species in this region (*3*). To determine the comparative species prevalence by multispecies rapid diagnostic test (RDT) and blood-smear microscopy, we conducted a cross-sectional survey of 631 children <5 years of age during the low transmission season of 2014 in 3 districts in southwestern Uganda (Mbarara, Bushenyi, and Isingiro) (Figure). These 3 districts represent a range of transmission intensities from low to high, respectively (*4*).

**Figure:**
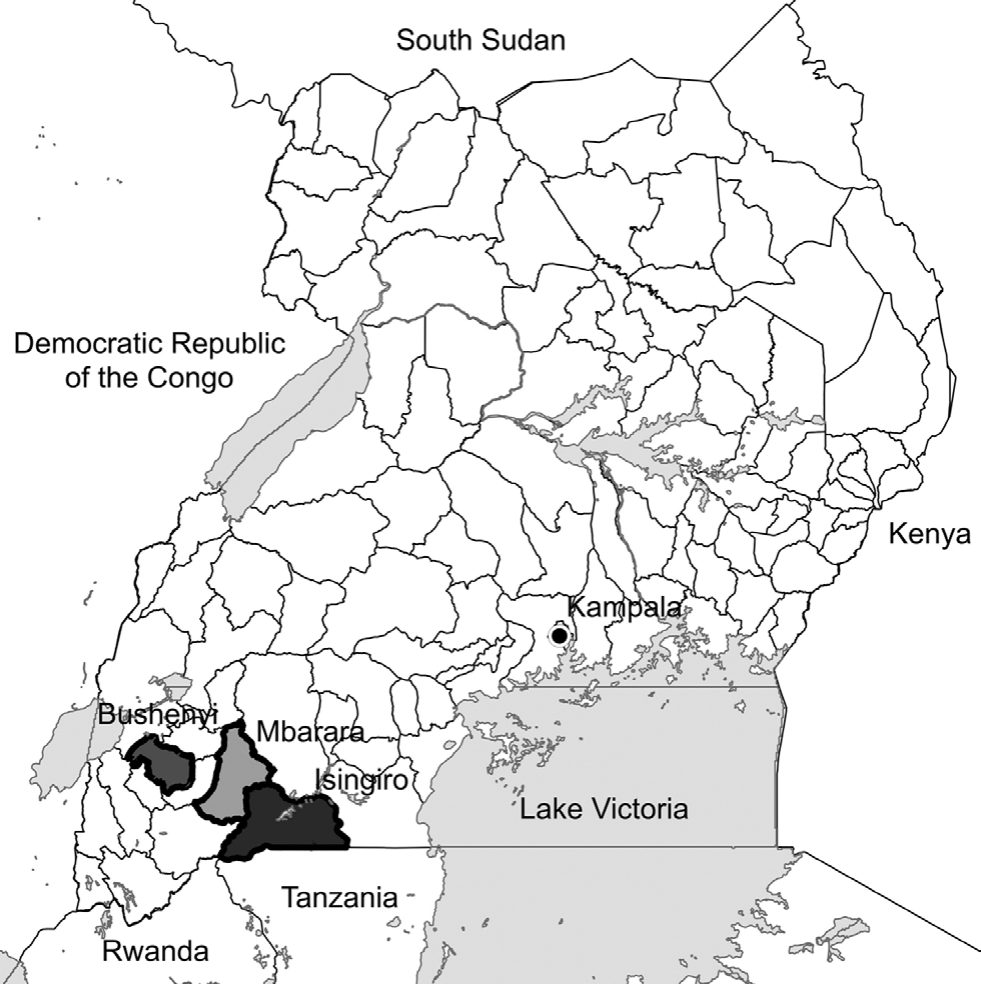
Districts where surveys of asymptomatic children were conducted to determine *Plasmodium* infections, southwestern Uganda.

## The Study

Stratified, 2-stage cluster sampling was used to select study participants. We administered questionnaires to gather information about standard knowledge, attitudes, and practices related to malaria and collected blood for testing with microscopy, RDT, and PCR (Technical Appendix). RDT was a combined *P. falciparum*–specific, histidine-rich protein-2 (HRP-2)/pan-*Plasmodium* lactate dehydrogenase (pLDH) RDT (SD Bioline Malaria Ag P.f/Pan [*P. falciparum* or other *Plasmodium* species]; Standard Diagnostics, Gyeonggi-do, South Korea). Nested PCR was performed on all RDT- or microscopy-positive samples. Predictors of malaria were selected a priori (online Technical Appendix).

We surveyed 631 children with a mean age of 2.4 years (Table 1). Bed net coverage was high (91.6%) and met targets for 2014 (*5*). Only 5 households (0.8% of children surveyed) reported use of indoor residual spraying. Of the 3 districts, Isingiro had the highest proportion of children living in the lowest wealth quartile (41.8%) and in households with thatched or leaf roofing (7.3%); this district also had the lowest consistent bed net use (85.7%) (Technical Appendix Table 1).

**Table 1:** Characteristics of asymptomatic children surveyed for *Plasmodium* infections, by district, southwestern Uganda*

Characteristic	**District†**				**p value‡**
	Mbarara, n = 242	Bushenyi, n = 157	Isingiro, n = 232	Total, N = 631†	
Mean age, y ± SD	2.4 ± 1.2	2.3 ± 1.2	2.4 ± 1.3	2.4 ± 1.3	0.850
Sex					
M	122 (50.4)	80 (51.0)	120 (51.7)	322 (51.0)	0.958
F	120 (49.6)	77 (49.0)	112 (48.3)	309 (49.0)	
Rural	177 (73.1)	133 (84.7)	202 (87.1)	512 (81.1)	0.472
Wealth quartile					<0.001
1st	42 (17.4)	45 (28.7)	97 (41.8)	184 (29.2)	
2nd	27 (11.2)	58 (36.9)	55 (23.7)	140 (22.2)	
3rd	83 (34.3)	32 (20.4)	56 (24.1)	171 (27.1)	
4th	90 (37.2)	22 (14.0)	24 (10.3)	136 (21.6)	
Roofing material					<0.001
Corrugated metal	237 (97.9)	152 (96.8)	206 (88.8)	595 (94.3)	
Thatch or leaf	3 (1.2)	5 (3.2)	17 (7.3)	25 (4.0)	
Other	2 (0.8)	0	9 (3.9)	11 (1.7)	
Household crowding§					0.239
1–2	68 (28.1)	52 (33.1)	61 (26.3)	181 (28.7)	
3	123 (50.8)	71 (45.2)	134 (57.8)	328 (52.0)	
>4	51 (21.1)	34 (21.7)	37 (16.0)	122 (19.3)	
Consistent bed net use	228 (94.2)	151 (96.2)	198 (85.7)	577 (91.6)	0.003
Indoor residual spraying	4 (1.7)	1 (0.6)	0	5 (0.8)	0.122
Malaria prevalence					
By RDT	4 (1.7)	8 (5.1)	30 (13.0)	42 (6.7)	<0.001
Pf+	1	3	7	11	
Pan+	2	0	1	3	
Pf/Pan+	1	5	22	28	
By microscopy	4 (1.7)	5 (3.2)	13 (5.6)	22 (3.5)	0.067
* P. falciparum*	2	3	4	9	
* P. malariae*	0	1	6	7	
* P. ovale*	1	1	1	3	
Mixed *Pf/Pm*	1	0	2	3	

*Values are no. (%) children surveyed except as indicated. Pan+, positive for non–*P.* *falciparum* infection only; Pf+, positive for *P. falciparum *monoinfection only; Pf/Pan+, positive for *P. falciparum* monoinfection or *P. falciparum* mixed infection; RDT, rapid diagnostic test; mixed *Pf/Pm*, positive for *P. falciparum *monoinfection or* P. malariae *mixed infection.  †Totals in columns may not add up to total because of missing data. ‡Determined by Fisher exact test or χ^2^ test, as appropriate.  §Defined as number of persons who sleep in the same room.

Overall prevalence of parasitemia by microscopy was 3.5% (95% CI 1.9%–5.1%). Speciation by microscopy revealed a higher proportion of non–*P. falciparum* infections than *P. falciparum* monoinfections. Of 22 microscopy-positive samples, 9 (40.9%) were *P. falciparum* monoinfections, 7 (31.8%) were *P. malariae* monoinfections, 3 (13.6%) were *P. ovale* monoinfections, and 3 (13.6%) were *P. falciparum*/*P. malariae* mixed infections. Most *P. malariae* monoinfections occurred in Isingiro district. Sixteen (72.7%) of 22 blood-smear readings correlated directly with PCR results (Technical Appendix Table 2).

Malaria parasite prevalence was 2-fold higher by RDT than by microscopy (6.7% vs. 3.5%; Table 1). RDT correctly identified 9 of 12 *P. falciparum* monoinfections and mixed infections. A comparison of the diagnostic performance of RDT and microscopy (uncorrected by PCR) indicates that agreement of results from these methods was high (>94%); however, agreement was poor in detecting non–*P. falciparum* infections (κ = 0.15) compared with detecting overall infection (κ = 0.41) and *P. falciparum* infection (κ = 0.33) (Table 2). PCR detected parasite DNA in 53.7% (22/41) of RDT-positive samples; of these, 55% (12/22) correlated with the correct RDT band pattern interpretation (Technical Appendix Table 2).

**Table 2:** Diagnostic performance of RDT and microscopy for *Plasmodium* infections in children in 3 districts, southwestern Uganda*

Diagnostic accuracy of RDT†	*Plasmodium* infection	*P. falciparum* infection	Non–*P. falciparum* infection‡
Sensitivity (95% CI)	63.6 (40.7–82.8)	75.0 (42.8–94.5)	10.0 (0.3–44.5)
Specificity (95% CI)	95.4 (93.4–96.9)	95.1 (93.1–96.7)	99.8 (99.1–100.0)
PPV (95% CI)	33.3 (19.6–49.5)	23.1 (11.1–39.3)	50.0 (1.3–98.7)
NPV (95% CI)	98.6 (97.3–99.4)	99.5 (98.5–99.9)	98.6 (97.3–99.3)
Agreement, %	94.3	94.8	98.3
κ	0.41	0.33	0.15
*NPV, negative predictive value; PPV, positive predictive value; RDT, rapid diagnostic test. †SD Bioline Malaria Ag Pf/Pan (*P. falciparum* or other *Plasmodium* species; Standard Diagnostics, Gyeonggi-do, South Korea). ‡Children with mixed infections were excluded from analysis.			

Approximately one third (8/22) of children with microscopy-positive cultures had negative RDT results (Technical Appendix Table 3). Of these 8 discordant cases, 5 harbored PCR-confirmed *P. malariae* or *P. ovale* monoinfections; all had parasite densities <1,060/μL (Technical Appendix Table 2). Conversely, two thirds (28/42) of RDT-positive samples were negative by microscopy. Of the 28 children with RDT-positive and microscopy-negative samples, 8 (28.6%) had a malaria infection within the previous month; 4 of those had detectable parasite DNA.

## Conclusions

Our findings indicate that strides in the control of *P. falciparum* malaria have continued in Uganda. Compared with data from 2010, *P. falciparum* prevalence by microscopy had a 4- and 5-fold decrease in urban and rural villages, respectively (*3*). Our estimates are consistent with prevalence estimates from 2009 (12%) and 2014–2015 (4%) (*1*,*6*).

In our study, 46% of asymptomatically infected children harbored non–*P. falciparum* species, particularly *P. malariae*, in contrast to the 1.2% non–*P. falciparum* species prevalence in 2009 (*6*). In addition, 1 *P. vivax* monoinfection was detected by PCR in Isingiro, confirming the continued presence of all 4 major species in Uganda (Technical Appendix Table 2) (*1*). Furthermore, although most *P. malariae* cases were from Isingiro, recent studies in other regions of Uganda (i.e., northern and eastern) have also reported a rise in non–*P. falciparum* infections, particularly *P. malariae* (*7*–*9*).

One possible reason for the nearly equivalent prevalence of asymptomatic *P. falciparum* and non–*P. falciparum* infections is the influence of seasonal fluctuations in species prevalence; for example, *P. malariae* prevalence has been higher during the dry season in West Africa (*10*). Another possibility is that the use of *P. falciparum*–based RDTs, which are advantageous because of low infrastructure costs and high prevalence of this species in Uganda, has enabled non–*P. falciparum* prevalence to go undetected. Alternatively, our results may represent a true shift in species prevalence. What is apparent is that pLDH/HRP-2–based RDTs may not be the most sensitive diagnostic method to determine true prevalence in the future. In our study, RDT was negative in all 3 microscopy-identified *P. ovale* and in 3 of 7 *P. malariae* monoinfections, a finding that may be in part attributable to these species’ low parasite densities (range 39–1,057/µL).

Identifying *P. malariae* and *P. ovale* infections is critical because *P. malariae* has been associated with chronic infections that can persist for years, including a chronic nephrotic syndrome that, once established, is unresponsive to treatment (*10*). Because these species have milder symptoms and lower parasite prevalence than *P. falciparum*, infections can remain undetected for extended periods, enabling persons to serve as reservoirs for ongoing transmission (*2*). These species may transmit gametocytes more efficiently at low parasite densities; a recent study found increased *P. falciparum* gametocyte production in the setting of mixed *P. malariae* infections (*11*). In our study, 30% of non–*P. falciparum* monoinfections harbored gametocytes. Finally, on the therapeutic side, studies have shown failure of parasite clearance after artemisinin-based combination therapy in non–*P. falciparum* infections, including *P. malariae* (*2*,*9*,*12*). *P. ovale* and *P. vivax* also form hypnozoites in the liver, and safe treatment with a 14-day course of primaquine is necessary to clear parasitemia. Six children in our study harbored *P. ovale* or *P. vivax* infections.

From a malaria control perspective, the performance of the pLDH/HRP-2–based RDT was suboptimal in our data, indicating a need for accurate diagnostic methods aimed at detecting *Plasmodium* infections in this region. A diagnostic method that has been effective in resource-constrained settings is loop-mediated isothermal amplification (LAMP), which affords higher sensitivity in detecting low-level parasitemia, especially *P. malariae* infections that tend to exhibit lower parasite densities than *P. falciparum* and *P. vivax* (*13*). In a 2013 rural Uganda study, the sensitivity of LAMP was ≈1.8-fold greater than microscopy, comparable to PCR (*14*). Wide-scale application of a field-friendly technique such as LAMP might be possible in southwestern Uganda, where asymptomatic persons might have low-density malaria infections that persist during the low malaria transmission season, enabling these persons to serve as reservoirs for ongoing transmission and disease (*15*). Effective methods for detecting and treating these infections are essential for controlling and eliminating malaria.

Technical Appendix Detailed methods and tables describing risk factors for asymptomatic malaria infections and results of PCR-corrected samples found to be positive by microscopy and rapid diagnostic test.
