# Supplementary material for: Asymptomatic Plasmodium Infections in Children in Low Malaria Transmission Setting, Southwestern Uganda
Source: Emerg Infect Dis. 2016 Aug;22(8):1494–8. doi: 10.3201/eid2208.160619 (PMC4982177; doi:10.3201/eid2208.160619)
Supplement: Technical Appendix — Detailed methods and tables describing risk factors for asymptomatic malaria infections and results of PCR-corrected samples found to be positive by microscopy and rapid diagnostic test. [file 16-0619-Techapp-s1.pdf]

Article DOI: http://dx.doi.org/10.3201/eid2208.160619

Asymptomatic *Plasmodium* Infections in Children in Low Malaria Transmission Setting, Southwestern Uganda

Technical Appendix

Methods

The study was conducted in southwestern Uganda, in an area with an annual entomologic inoculation rate of 2–4 infective bites per year, and biannual rainy seasons. The survey occurred during August–October 2014, corresponding to the low transmission season. A stratified, two-stage cluster sampling design was used to select participants. In the first sampling stage, 60 villages were sampled (20 from each of 3 districts) and stratified on the basis of their urban or rural status. Villages were sampled by using probability-proportionate-to-size sampling. Within each village, households were randomly selected by using the World Health Organization's Expanded Program on Immunization methodology. The number of households selected in each village was weighted on the basis of the population size of each district. In each household, only 1 child <5 years of age who met the eligibility criteria was randomly selected to participate in the survey. If no children were in the household, the field team would move on to the next house until the total number of households required for each village was met. Allowing for a 10% nonresponse rate and a design effect of 1.5, a sample size of 601 children had 80% power to detect 5% difference in malaria prevalence from that determined in the 2009 Uganda Malaria Indicator Survey (12% in southwestern Uganda) (*1*).

Field Procedures and Sample Collection

After informed parental consent was obtained, standardized questionnaires were administered to the head of household or parent or guardian as face-to-face interviews to collect information about their demographics and malaria control measures. Information included consistent bed net use, household wealth, household construction, household crowding, district, and rural or urban status of residence. We collected ≈250 µL of blood by fingerprick into an

EDTA-coated microtainer (BD Diagnostics, Franklin Lakes, NJ, USA). Rapid diagnostic tests (RDTs) were performed, and thick and thin smears for microscopy were prepared on-site by trained laboratory technicians. In sets of 3 from participants, 50 μL of blood was spotted onto filter paper (Whatman 903 Protein Saver Card, Sigma-Aldrich, St. Louis, MO, USA) and stored at –80°C until shipment to the Yale School of Public Health (New Haven, CT, USA). QIAamp DNA Mini Kit (Qiagen, Valencia, CA, USA) was used to extract DNA from dried blood spots. A total of 100 μL was eluted from each sample.

Parasite Detection

Parasite detection was assessed by using a combined *P. falciparum*–specific histidine-rich protein-2 (HRP-2)/pan-*Plasmodium* lactate dehydrogenase (pLDH) RDT (SD Bioline Malaria Ag Pf/Pan, Standard Diagnostics, Gyeonggi-do, Republic of Korea) and microscopy. RDT was performed according to manufacturer's instructions. If the control line of the RDT did not appear, the test was considered invalid and was repeated. A positive Pf-HRP-2 band indicated a *P. falciparum* infection, whereas a positive pan-pLDH band alone represented an infection with ≥1 non–*P. falciparum* species. A result with both the Pf-HRP-2 and pan-pLDH bands indicated a *P. falciparum* monoinfection or a mixed *P. falciparum*/non–*P. falciparum* infection. For microscopy, smears stained with 10% Giemsa (pH 7.2) were read independently by 2 trained microscopists, with discordant results resolved by a third reader. Parasite and gametocyte densities, expressed as number of parasites/μL, were calculated by counting parasite numbers against 200 leukocytes and multiplied by an assumed standard leukocyte count of 8,000 leukocytes/μL, as recommended by the World Health Organization (*2*).

For all patients with either a positive microscopy or RDT result, species confirmation by nested PCR was performed for *Plasmodium falciparum, P. vivax, P. ovale,* and *P. malariae*. Primers were complementary to the *Plasmodium* small subunit ribosomal DNA gene as previously described by Singh et al (*3*). For both the primary and nested rounds of amplification, PCR was performed by using 5X GoTaq Flexi Buffer (Promega, Madison, WI, USA), 1.5 mmol/L magnesium chloride, 0.2 μmol/L deoxyribonucleotide triphosphate, 0.2 μmol/L of forward and reverse primers, 1 U GoTaq polymerase (Promega), and 2 μL template DNA. Thermocycling conditions were as follows: 94°C for 1 min, followed by 35 and 30 cycles of 94°C for 1 min, 58°C for 2 min, 72°C for 5 min for the primary and nested rounds, respectively, and a final extension of 72°C for 5 min. Positive controls were acquired from Malaria Research

and Reference Reagent Resource Center (MR4, Biodefense and Emerging Infections Resources Repository, National Institute of Allergy and Infectious Diseases, National Institutes of Health, Manassas, VA, USA). Products of nested PCR were analyzed by gel electrophoresis.

Statistical Analysis

Data was analyzed by using Stata 14.0 (StataCorp, College Station, TX, USA). Categorical variables were compared by using the χ^2 test or Fisher exact test, as appropriate. T-tests were performed when comparing 2 groups. Wilcoxon rank-sum tests were used when continuous variables were nonparametric. If the variables contained >2 independent groups, 1-way ANOVA was performed to calculate the difference between means. All statistical tests accounted for our clustered sampling design.

To assess the diagnostic performance of the SD Bioline Malaria Ag P.f/Pan (*P. falciparum* and other *Plasmodium* species) RDT (Standard Diagnostics, Gyeonggi-do, South Korea), results of RDT and microscopy were treated as binary (i.e., positive or negative) outcomes, and microscopy was considered the gold standard. Sensitivity, specificity, and negative and positive predictive values were calculated to assess RDT performance in diagnosing infection with any species, *P. falciparum* infection, and non–*P. falciparum* infection. Mixed infections with *P. falciparum* were excluded from the analysis only when assessing RDT performance in diagnosing non–*P. falciparum* infections. The kappa statistic was calculated to measure agreement between RDT and microscopy results.

Predictor variables were selected *a priori* based on their individual, household, or environmental risk for malaria infection. Predictors of interest were consistent bed net use, household wealth, household building materials, household crowding, district of residence, and rural or urban status of the village in which the participant resided. Consistent bed net use (a binary variable) was defined as a participant who reported always sleeping under a bed net. Household wealth quartiles were constructed by using a multicomponent analysis of household possessions, household roof and flooring material, and source of drinking water. For assessing predictors of positive microscopy cases, Poisson regression with robust variance was used to calculate bivariate and multivariate prevalence ratios. Predictors that were statistically significant at the 0.10 level in the univariate analysis were included in the multivariate model.

Ethics Approval

Written informed consent for study participation was obtained from the children's parent or guardian. Ethical approval was obtained from the Uganda National Council of Science and Technology, the Mbarara University of Science and Technology Institutional Review Council, and the Yale University Human Investigation Committee.

References

1. Uganda Bureau of Statistics (UBOS) and ICF Macro. Uganda malaria indicator survey 2009. 2010 Aug [cited 2014 March 24]. http://dhsprogram.com/pubs/pdf/MIS6/MIS6.pdf.

2. World Health Organization. Basic malaria microscopy: part II. Tutor's guide. 2010 [cited 2016 Jun 10]. http://apps.who.int/iris/bitstream/10665/44208/2/9789241547918_eng.pdf?ua=1&ua=1

3. Singh B, Bobogare A, Cox-Singh J, Snounou G, Abdullah MS, Rahman HA. A genus- and species-specific nested polymerase chain reaction malaria detection assay for epidemiologic studies. Am J Trop Med Hyg. 1999;60:687–92.

Technical Appendix Table 1. Factors associated with asymptomatic parasitemia detectable by microscopy*

Variable	Unadjusted PR	(95% CI)	p value†	Adjusted PR	(95% CI)	p value†
Location of residence						
Urban	1.00	Ref				
Rural	2.33	(0.58-9.37)	0.229			
District of residence						
Mbarara or Bushenyi	1.00	Ref		1.00	Ref	
Isingiro	2.48	(0.97-6.34)	0.058	1.81	(0.68-4.83)	0.229
Wealth quartile						
1st–3rd	1.00	Ref		1.00	Ref	
4th	0.17	(0.24-1.23)	0.079	0.33	(0.05-2.35)	0.266
HH roofing material						
Corrugated metal/other	1.00	Ref		1.00	Ref	
Thatch/leaf	7.12	(2.56-19.76)	<0.001	5.13	(1.72-15.31)	0.004
HH flooring material						
Cement	1.00	Ref		1.00	Ref	
Earth/dirt	2.96	(0.91-9.67)	0.071	1.38	(0.49-3.97)	0.533
HH crowding						
<3 person/room	1.00	Ref		1.00	Ref	
≥3 person/room	1.94	(0.92-4.11)	0.081	1.76	(0.78-3.96)	0.168
Bed net use						
Inconsistent	1.00	Ref		1.00	Ref	
Consistent	0.31	(0.13-0.74)	0.009	0.40	(0.19-0.83)	0.016

*HH, household; PR, prevalence ratio; Ref, reference.
†p values determined χ^2 test or Fisher exact test, as appropriate.

Technical Appendix Table 2. PCR confirmation of microscopy-positive and RDT-positive results*

		Nested PCR results						
Test type	Type of infection	Negative	*P. falciparum*	*P. malariae*	*P. ovale*	*P. vivax*	Mixed	Total
Microscopy, n = 22	*P. falciparum*	0	6	0	2	0	1†	9
	P. malariae	0	0	6	0	0	1‡	7
	P. ovale	1	0	0	2	0	0	3
	Mixed§	0	0	1	0	0	2§	3
	Total	1	6	7	4	0	4	22
RDT¶, n = 41	Pf-band+	6	3	0	0	1	1§	11
	Pan-band+	0	0	2	0	0	1#	3
	Pf/Pan-bands+	13	6	3	3	0	2‡,	27
	Total	19	9	5	3	1	4	41

*Pan-band+, positive for non–*P. falciparum* infection only; Pf-band+, positive for *P. falciparum* monoinfection only; Pf/Pan-bands+, positive for *P. falciparum* monoinfection **or** *P. falciparum* mixed infection; RDT, rapid diagnostic test.
†Mixed infection of *P. falciparum, P. malariae, P. ovale*, and *P. vivax*.
‡Mixed infection of *P. malariae, P. ovale*, and *P. vivax*.
§Mixed infection of *P. falciparum* and *P. malariae*.
¶1 RDT result did not have an available blood sample.
#Mixed infection of *P. ovale* and *P. vivax.*

Technical Appendix Table 3. Results of RDT and microscopy for malaria species infections in 631 children in 3 districts in southwestern Uganda*

	Light microscopy results					
Rapid diagnostic test results	Pf+	Pm+	Po+	Pf/Pm+	Negative	Total
Pf+	1	0	0	1	9	11
Pan+	0	1	0	1	1	3
Pf/Pan+	6	3	0	1	18	28
Negative	2	3	3	0	579	587
Total	9	7	3	3	607	629†

*Pan+, positive for non–*P. falciparum* infection only; Pf+, positive for *P. falciparum* monoinfection only; Pf/Pan+, positive for *P. falciparum* monoinfection or *P. falciparum* mixed infection; Pf/Pm+, positive for *P. falciparum* and *P. malariae* mixed infection; Pm, positive for *P. malariae* infection; Po, positive for *P. ovale* infection; RDT, rapid diagnostic test.
†RDT data is missing for 1 child; microscopy data is missing for 1 child.
